# Heart-specific Rpd3 downregulation enhances cardiac function and longevity

**DOI:** 10.18632/aging.100806

**Published:** 2015-09-21

**Authors:** Zachary A. Kopp, Jo-Lin Hsieh, Andrew Li, William Wang, Dhelni T. Bhatt, Angela Lee, Sae Yeon Kim, David Fan, Veevek Shah, Emaad Siddiqui, Radhika Ragam, Kristen Park, Dev Ardeshna, Kunwoo Park, Rachel Wu, Hardik Parikh, Ayush Parikh, Yuh-Ru Lin, Yongkyu Park

**Affiliations:** ^1^ Department of Cell Biology and Molecular Medicine, Rutgers-New Jersey Medical School, Newark, NJ 07103; USA

**Keywords:** Rpd3, cardiac function, stress resistance, aging, gene expression

## Abstract

Downregulation of Rpd3, a homologue of mammalian Histone Deacetylase 1 (HDAC1), extends lifespan in *Drosophila melanogaster*. Once revealed that long-lived fruit flies exhibit limited cardiac decline, we investigated whether Rpd3 downregulation would improve stress resistance and/or lifespan when targeted in the heart. Contested against three different stressors (oxidation, starvation and heat), heart-specific Rpd3 downregulation significantly enhanced stress resistance in flies. However, these higher levels of resistance were not observed when Rpd3 downregulation was targeted in other tissues or when other long-lived flies were tested in the heart-specific manner. Interestingly, the expressions of anti-aging genes such as *sod2*, *foxo* and *Thor*, were systemically increased as a consequence of heart-specific Rpd3 downregulation. Showing higher resistance to oxidative stress, the heart-specific Rpd3 downregulation concurrently exhibited improved cardiac functions, demonstrating an increased heart rate, decreased heart failure and accelerated heart recovery. Conversely, Rpd3 upregulation in cardiac tissue reduced systemic resistance against heat stress with decreased heart function, also specifying phosphorylated Rpd3 levels as a significant modulator. Continual downregulation of Rpd3 throughout aging increased lifespan, implicating that Rpd3 deacetylase in the heart plays a significant role in cardiac function and longevity to systemically modulate the fly's response to the environment.

## INTRODUCTION

As a histone deacetylase (HDAC), Rpd3 modulates chromatin structures, including the heterochromatin of *Drosophila* telomeres, by interacting with several chromatin remodeling complexes [1]. The Rpd3 protein is also reported to mediate epigenetic effects like long-term memory and lifespan. Overexpression or RNAi-mediated knockdown of Rpd3 in the adult fly brain results in impaired long-term courtship memory [2]. However, systemic Rpd3 downregulation extends lifespan in *rpd3*−/+ heterozygous mutants [3] although *rpd3*−/− homozygotes are lethal. This longer lifespan is also detected in yeast with Rpd3 downregulation [4]. Consistently, feeding *Drosophila* 4-phenylbutyrate (PBA, an inhibitor of histone deacetylase) throughout adulthood could significantly increase lifespan [5]. Several long-lived mutant flies have displayed increased resistance to numerous stressors including oxidation, starvation and heat over wild-type flies, indicating a positive correlation between stress resistance and lifespan extension [6-9]. Downregulation of Rpd3 has been known to extend lifespan in *Drosophila* [3], however its relationship to stress resistance has not yet been characterized.

Heart function declines in aging fruit flies, exhibiting that heart rate decreases and instead stress-induced heart failure increases [10]. Such age-related changes are minimized in long-lived flies when systemic levels of Insulin-IGF receptor (InR) signaling are reduced. Interestingly, interfering with InR signaling exclusively in the heart prevents the decline in cardiac performance with age [10]. Thus, the fly heart is shown to be a reliable age-related cardiac disease model for studying age-dependent decline in organ function [11]. Mammalian Sirtuins, class III histone deacetylases, are reported to have protective and beneficial effects against numerous age-related diseases, including cardiovascular pathologies [12]. Non-sirtuin histone deacetylases (Classes I, II and IV HDACs), are also known to serve a role in controlling cardiac aging. HDAC1, a mammalian homolog of Rpd3, has crucial roles in heart development and physiology [13], in which phosphorylation of HDAC1 promotes enzymatic activity and complex formation [14-16].

Here, we provide that decreased Rpd3 expression in *Drosophila* heart tissue enhances cardiac functions and stress resistances against three different stressors (oxidation, starvation and heat) with systemically increased expression of anti-aging genes such as *sod2*, *foxo* and *Thor*, while showing no effect from other tissue-specific Rpd3 modulations. Conversely, increased Rpd3 expression in the heart decreases resistance to heat stress and heart function when under stress, also representing that Rpd3 phosphorylation levels in the heart are related to modulation of cardiac function and stress resistance systemically. Finally, we show that lifespan is extended when Rpd3 is downregulated in the heart continuously during aging.

## RESULTS

### Rpd3 downregulation enhances resistance to oxidative stress

**Figure 1:**
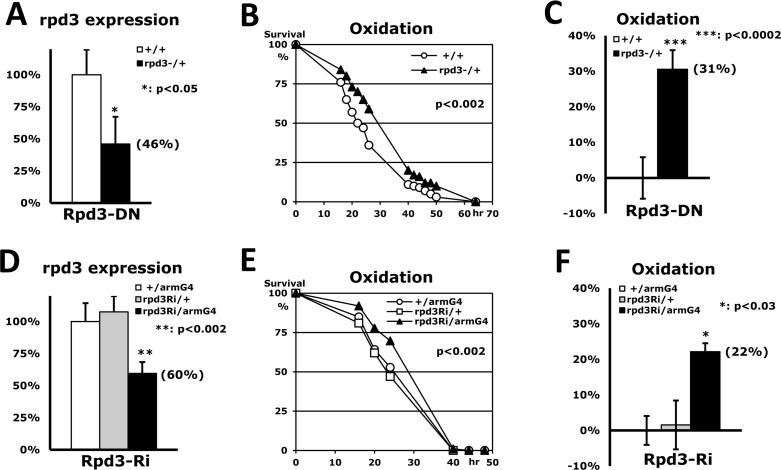
Stress resistance induced by Rpd3 downregulation (**A**) Expression level of the *rpd3* gene between 2-day-old adult male flies of wild-type (+/+: 100%) and *rpd3* heterozygous mutant (*rpd3*−/+) using RT-PCR. Bar: mean ± standard error of the mean (SEM) from six independent experiments with three different cDNAs; parenthesis: expression level changed; p-value (*): Student's t-test. (**B**) The difference in oxidative stress tolerance between 2-day-old wild-type male flies and the *rpd3* mutants. The survival curve (p-value: log-rank test) indicates that oxidative resistance is significantly enhanced by reduced *rpd3* expression. (**C**) The median survival times of the flies were calculated from the survival curves (**B**) of six independent experiments and then averaged for their mean ± SEM. The increased percentage (parenthesis) of the *rpd3* mutant's median survival time is represented following normalization with the wild-type's median (21.4 hours). (**D**) Expression level of the *rpd3* gene between two single transgene controls (+/armG4, rpd3Ri/+) and the double transgene experimental groups (rpd3Ri/armG4) from twelve independent experiments. (**E**) The difference in oxidative stress tolerance between the two controls and the experimental group. The p-value represents the comparison between the controls and rpd3Ri/armG4. (**F**) The median survival times of the flies were calculated from the survival curves (**E**) of four independent experiments and then normalized with the median of the control (*+*/armG4: 22.7 hours).

To investigate whether Rpd3 is related to the mechanism of stress resistance, a long-lived *rpd3* heterozygous (P{PZ}rpd3[04556]/+) mutant fly [3] was incubated with paraquat-induced oxidative stress. Although *rpd3*−/− homozygous mutant flies are lethal, we found that the *rpd3*−/+ heterozygous flies (46% of *rpd3* expression in Fig. 1A) can increase survivorship up to 31% under the oxidative stress compared to wild type (+/+) flies (Fig. 1B-C). This indicates that the Rpd3 downregulation enhances stress resistance as shown in other long-lived flies such as *mth* or *loco* heterozygous mutant flies [6, 17]. The lifespan of this Rpd3 downregulation was extended consistent with the previous report [3] although the content of extension (13% in SFig. 1A-B) was less than the previous observation (33% in [3]), which might be explained due to the different genetic background [18].

In order to validate our findings, we utilized the UAS/Gal4 system [19] which provides an alternate approach to reinvestigate if the Rpd3 downregulation enhances stress resistance. The *rpd3* expression was reduced via a UAS-*rpd3*-dsRNAi transgene (hereafter rpd3Ri in Fig. 1D) under the *arm*- or *act*-Gal4 driver, which respectively expresses a target UAS-gene mildly or highly in a whole-body manner. The highly downregulated *rpd3* expression in rpd3Ri/actG4 flies induced lethality as shown in the *rpd3*−/− homozygous mutant flies. However, the rpd3Ri/armG4 flies exhibited 60% of *rpd3* expression compared to the single transgene control (+/armG4 in Fig. 1D) and increased survivorship up to 22% under the oxidative stress (Fig. 1E-F). This mild *rpd3* downregulation of rpd3Ri/armG4 flies also extended lifespan (16% in SFig. 1C-D), implying that the mild decrease of *rpd3* expression in the whole body is favorable for enhancement of both stress resistance and lifespan.

### Heart-specific downregulation of Rpd3 increases stress resistance

**Figure 2:**
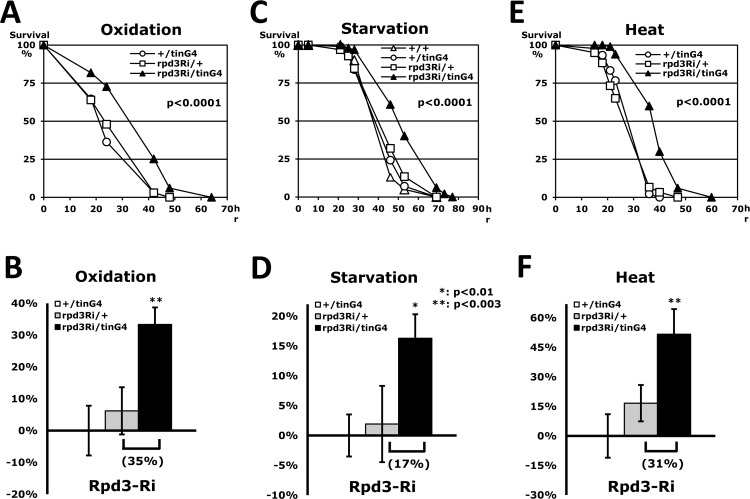
Stress resistance induced by heart-specific Rpd3 downregulation (**A, C, E**) Tolerance against oxidation (**A**), starvation (**C**), and heat stressors (**E**) between 2-day-old male flies of wild-type (+/+), single transgene controls (+/tinG4 and rpd3Ri/+) and heart-specific Rpd3 downregulation (rpd3Ri/tinG4). The survival curves show statistical significance in p-values (log-rank test), indicating that resistance to each stressor is enhanced with reduced *rpd3* expression in the heart. (**B, D, F**) The percent change in fly median survival time against the three stressors is shown as mean ± SEM that were calculated from the stress response curves (**A, C, E**) of 7 - 18 independent experiments. The data represents percentages (%) normalized from the +/tinG4 genotype medians (oxidation: 26.1; starvation: 36.6; heat: 21.1 hours).

Wessells *et al*. (2004) [10] reported that age-related change of heart function is minimized in long-lived flies when systemic levels of Insulin-IGF receptor (InR) signaling are reduced. Moreover, interfering with InR signaling exclusively in the heart prevents the decline in cardiac performance with age. According to those observations, we investigated if heart-specific Rpd3 downregulation affects heart function, stress resistance, and lifespan. Using a heart-specific driver (tinman-Gal4) [20], we examined the effect of Rpd3 downregulation (rpd3Ri) under three different stressors (oxidation, starvation and heat in Fig. 2). It was confirmed that the tinman-Gal4 driver (tinG4) displays the GFP signal in a heart-specific pattern (long heart tube from posterior abdomen in SFig. 2A) and expresses a target UAS-GFP gene that can be detectable in total RNAs purified from a whole-body (SFig. 2B). Although the change of rpd3 expression could not be detected with the heart-specific Rpd3 downregulation (rpd3Ri/tinG4) from the total RNAs of whole-body (data not shown), the rpd3Ri/tinG4 male flies interestingly revealed a 35% increase of the median survival time (hour) in resistance against the oxidative stress (Fig. 2A and D). Similarly, starvation and heat resistances were also enhanced by 17% and 31%, respectively, on average median over single transgene controls (Fig. 2). All three assays were statistically significant (P < 0.01), which exhibit that the heart-specific Rpd3 downregulation improves resistance to all of the stress tests: oxidation, starvation and heat. These enhancements of stress resistance could be generally exhibited in female flies as well (SFig. 3). We found that the heart-specific Rpd3 downregulation in females also improves stress resistance significantly in response to oxidation and starvation stressors, accumulating survival times approximately 47% and 60% longer than the single transgene control, respectively (SFig. 3). Surprisingly, these increased yields in females were higher than those of male flies (Fig. 2), indicating that the stress resistance is more effectively enhanced in females by heart-specific Rpd3 downregulation. However, in contrast to males, it appeared that female tolerance to heat did not increase significantly (data not shown), which may be interpreted as a gender difference. All together these findings indicate that the tissue-specific regulation of Rpd3 can systemically modulate the fly's response to the environment.

### Stress resistance is enhanced exclusively by heart-specific Rpd3 downregulation

**Figure 3:**
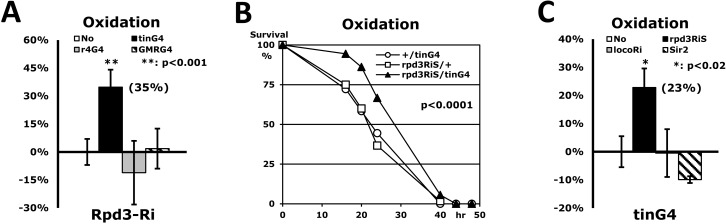
Only heart-specific Rpd3 downregulation enhances stress resistance (**A**) Rpd3 downregulation (rpd3Ri) limited to particular tissues (No: rpd3Ri/+ control; tinG4: heart; r4G4: fatbody; GMRG4: eye) on an oxidation stress assay. 2-day-old male flies were used for this experiment, with the exception of the rpd3Ri/r4G4 genotype, which used 5-day-old females due to the semi-lethality of the males. The percent change in median survival time of the flies is indicated as mean ± SEM, which was calculated from the stress response curves of at least 4 independent experiments. The data represent percent differences (%) normalized from the rpd3Ri/+ control (No), indicating that only heart-specific Rpd3 downregulation (rpd3Ri/tinG4) exhibits a statistically significant impact on stress tolerance (p <0.001). (**B**) The oxidation stress survival curve of heart-specific Rpd3 downregulation using a different rpd3RNAi (rpd3RiS/tinG4). The target region of rpd3Ri: 482bp in the middle of exon; rpd3RiS: 21bp in the 3′ UTR. (**C**) Heart-specific modulation of long-lived genes (No: +/tinG4 control; rpd3RiS: Rpd3 downregulation; locoRi: Loco downregulation; Sir2: Sir2 upregulation) on an oxidation stress assay. The percent change of median survival times was calculated from the 3 - 5 independent curves (**B**) of the oxidative stress test. The percentage data, normalized from the +/tinG4 control (No), shows that within the heart only Rpd3 downregulation (rpd3Ri/tinG4) significantly enhances stress resistance. The decrease of stress resistance in the rpd3Ri/r4G4 (**A**) and Sir2/tinG4 (**C**) genotypes were not statistically significant when compared to their respective single transgene controls (+/r4G4 and Sir2/+).

The Rpd3 gene is ubiquitously expressed in all fly tissues (http://flybase.org), implying that the Rpd3 may perform an important role everywhere as histone deacetylase. Accordingly, we investigated if Rpd3 down-regulation in other non-cardiac tissues also enhances stress resistance systemically as it did in the heart tissue (Fig. 3A). The fat body, the fruit fly analogue of mammalian liver and adipose tissues, is known to function in metabolic homeostasis, stress tolerance, growth and longevity in *Drosophila* [21, 22]. The fat body-specific modulation of a specific gene, such as overexpressed dFOXO, increases stress resistance and lifespan in flies [23, 24]. It is also reported that activation of JNK (Jun-N-terminal Kinase)-NLaz (Neural Lazarillo) signaling in the fat body promotes stress tolerance and extends lifespan as well [22]. The Rpd3 downregulation in fat body (rpd3Ri/r4G4), however, caused the semi-lethality in male flies (data not shown) and did not enhance stress resistance in females (Fig. 3A). Similarly, eye-specific Rpd3 downregulation (rpd3Ri/GMRG4) also could not affect the stress resistance (Fig. 3A) without any defect of eye morphology (data not shown). Although limited tests were performed, only heart-specific downregulation of the *rpd3* gene enhanced the stress resistance (Fig. 3A), implying that the Rpd3 protein functions in the heart for the regulation of stress resistance.

To exclude a possibility of non-specific RNA interference from 482bp target sequences of rpd3Ri transgene, the specific 21bp of another region in the *rpd3* gene was tested as a new heart-specific dsRNAi (rpd3RiS/tinG4 in Fig. 3B). Although the 23% increase in stress resistance of the rpd3RiS/tinG4 flies (Figs. 3B-C) was less than the 35% increase of the rpd3Ri/tinG4 flies (Fig. 3A), it confirmed that dsRNAi of any region of the *rpd3* gene in the heart enhances the stress resistance systemically (Fig. 3). We also tested whether other long-lived flies can enhance heart-specific stress resistance (Fig. 3C). It has previously been found that the Loco downregulation throughout the entire body increases not only the resistance to three stressors, including oxidation (26-63% increase), but also extends lifespan (17-32% extension) [17]. However, Loco downregulation limited exclusively to the heart (locoRi/tinG4) had no effect on oxidative stress resistance (−0.5% in Fig. 3C), suggesting that the *rpd3* gene is specific for heart-specific stress resistance. Sir2, another histone deacetylase, was also investigated in a heart-specific manner. It was reported that the Sir2 upregulation extends lifespan in flies, but is dependent upon the Sir2 dosage [25]. Stress resistance, however, was not enhanced by either mild or high Sir2 upregulation in the heart (Sir2/tinG4 in Fig. 3C), implying that the Rpd3's deacetylase activity is directly related to the stress resistance mechanism within the heart tissue. Although the fat body-specific dFOXO upregulation increases stress resistance and lifespan in flies [23, 24], a heart-specific dFOXO upregulation could not be tested in stress resistance due to semi-lethality in our genetic background (data not shown), further implicating that the heart-specific Rpd3 exclusively contributes to the enhanced stress resistance.

### Heart-specific Rpd3 downregulation systemically increases expressions of anti-aging genes

**Figure 4:**
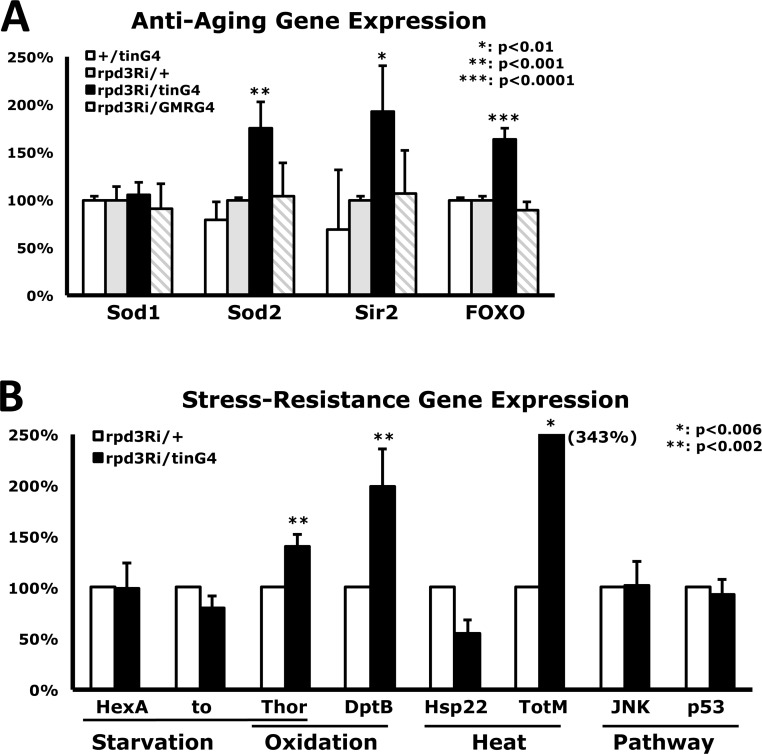
Changes in gene expression from heart-specific Rpd3 downregulation (**A**) Differential expression of anti-aging genes. Standardized against the rpd3Ri/+ single transgene control, comparative C_T_ analyses (RT-PCR) were performed using total RNAs purified from 2-day-old flies where *rpd3* was downregulated in the heart (tin) or eye (GMR). The bars represent a mixture of male and female data, which exhibited similar trends in 7 - 13 independent experiments with 4 - 5 different cDNAs. (**B**) Differential expression levels of representative stress-response genes between the rpd3Ri/+ control and heart-specific Rpd3 downregulation (rpd3Ri/tinG4), which are grouped by the specific stressor they are responsible for. The data was analyzed using 4 - 7 independent RT-PCR experiments from the total RNAs of 2-day-old male flies.

To characterize how heart-specific Rpd3 down-regulation systemically enhances stress resistance, expression of representative anti-aging genes was examined using total RNAs purified from whole flies. Although *sod1* (Cu/ZnSOD) expression did not change with heart-specific Rpd3 downregulation, *sod2* (MnSOD) expression increased by 75% in comparison with control rpd3Ri/+ flies (Fig. 4A), which may explain how heart-specific Rpd3 downregulation more efficiently removes the superoxide produced from paraquat in mitochondria. This increase of SOD expression was observed in several long-lived flies such as InR or Loco downregulated flies [17, 26]. It is reported that the long-lived *rpd3*−/+ mutants show a 134% increase in *sir2* expression and are related to caloric restriction [3]. Interestingly, heart-specific Rpd3 downregulation still resulted in increased expression of *sir2* gene by 93% (Fig. 4A). In *Drosophila*, moderate increase of Sir2 (2-5 folds) extends lifespan, although higher levels of Sir2 decrease the lifespan with a cellular toxicity [25]. When expression of *foxo*, another anti-aging gene, was measured, the heart-specific Rpd3 downregulation also increased the *foxo* expression by a significant 63% (Fig. 4A). It was reported that the dFOXO upregulation in brain fat body induces oxidative stress resistance and extends lifespan in flies, and is related to insulin signaling [23, 24]. These increased expressions of anti-aging genes did not result from Rpd3 downregulation in other tissues such as eye tissue (rpd3Ri/GMRG4 in Fig. 4A), indicating that only heart-specific Rpd3 downregulation systemically induces a significant increase of the anti-aging genes.

Furthermore, with heart-specific Rpd3 downregulation, the transcriptional level of representative stress-related genes was measured, which were selected from previously published stress response genes (http://flybase.org) [27, 28]. Expressional changes of Hexokinase A (*HexA*) and *takeout* (*to*) genes were not detected (Fig. 4B) although those proteins are related to starvation-tolerance mechanisms. However, the *Thor* gene (d4E-BP: eIF4E-binding protein) that mediates starvation and oxidative stress resistances was upregulated up to 40%, significantly (p<0.002 in Fig. 4B) with the heart-specific Rpd3 downregulation. This may partially explain how heart-specific Rpd3 downregulation can enhance resistance against starvation and oxidative stressors (Fig. 2 and SFig. 3). During oxidative stress and aging, antimicrobial peptide (AMP) genes were induced [28]. One of AMP genes, DptB, was 99% upregulated with the heart-specific Rpd3 downregulation (Fig. 4B). Heat shock protein (*Hsp*) and Turandot (*Tot*) genes are transcriptionally upregulated during heat stress and overexpression of *TotA* increases stress resistance in flies [28, 29]. Interestingly, heart-specific Rpd3 downregulation exhibited 343% expression of *TotM* compared to the control (Fig. 4B). However, heart-specific Rpd3 downregulation did not change gene expression of *JNK* and *p53* in stress responsive signaling pathway (Fig. 4B) although their protein activities have not yet been tested.

### Heart-specific Rpd3 downregulation enhances resistance against oxidative stress with higher cardiac function

Having observed increased stress resistance from heart-specific Rpd3 downregulation, we performed further experiments to determine whether there was an observable physiological change in cardiac function.

**Figure 5:**
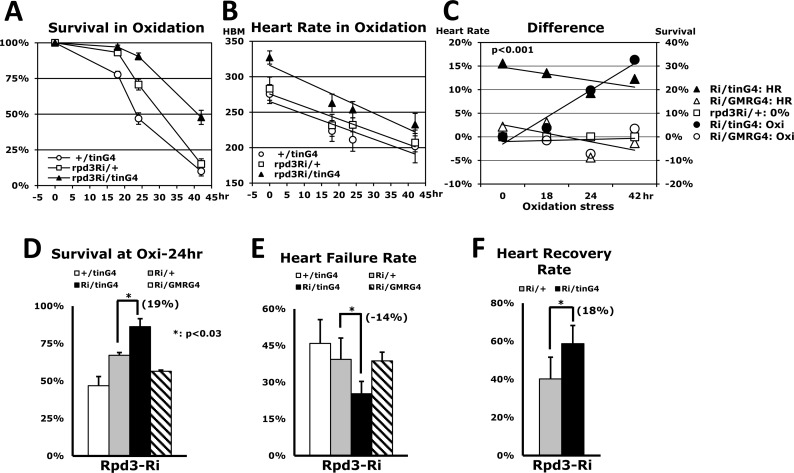
Overall heart function is enhanced by heart-specific Rpd3 downregulation (**A**) Oxidative stress tolerance between the 2-day-old male flies of two of single transgene controls (+/tinG4, rpd3Ri/+) and heart-specific Rpd3 downregulation (rpd3Ri/tinG4), which was made with the mean ± SEM over two independent assays. The survivorship (**A**) and heart rate (**B**) were measured across the same time points (0, 18, 24, and 42 hours). (**B**) The corresponding heart rates during the aforementioned oxidative stress test (**A**). More than 20 heart rates were measured at each time point. HBM: heart beat per minute. (**C**) Comparison of heart- and eye-specific Rpd3 downregulation (four independent assays). **Left**: The percent change of heart rate from the common control rpd3Ri/+ (0%) at each time point, which was calculated from the heart rate graphs (**B**). Only heart-specific Rpd3 downregulation displayed a statistically significant cardiovascular enhancement (p < 0.0001) compared to eye-specific downregulation (p = 0.94) from the control (rpd3Ri/+). **Right**: The differences in survivorship (%) for the oxidative stress test when compared to the common control rpd3Ri/+ (0%). The percentages were calculated from the four different time points (p = 0.03 - 0.0001) of the survival curves (**A**). (**D**) Comparison of oxidative stress resistance after 24 hours paraquat treatment among the +/tinG4, Rpd3Ri/+ single transgene controls and heart-specific (rpd3Ri/tinG4), eye-specific (rpd3Ri/GMRG4) Rpd3 downregulations. The survivorship percentage (%) at 24 hours was assessed as mean ± SEM over 5 - 7 independent stress assays. (**E**) The heart failure rate measured at 24 hours of oxidative stress (**A**) in 3 - 8 repeat experiments using a total of 22 - 134 flies. (**F**) The heart recovery rate measured at 24 hours of oxidative stress (**A**) in 5 repeat trials with a total of 66 - 67 flies.

The *Drosophila* heart exhibits physiological changes, such as lower heart rate, as it ages [10]. Interestingly, long-lived flies reducing an InR signaling have been shown to eliminate these manifestations of the aging process, prolonging younger cardiac function into old age as keeping higher heart rate [10]. We measured the heart rate of flies concurrent with paraquat-induced oxidative stress in the different time points: 0, 18, 24, and 42 hours (Figs. 5A-B). Our findings indicated that heart-specific Rpd3 downregulation (Rpd3Ri/tinG4) displays a gradual enhancement of oxidative stress resistance as a stressed period increases (Figs. 5A and C). The Rpd3Ri/tinG4, which yielded a 91% survival rate at 24 hours, was at least 20% higher than the survival rates for both single transgene controls: rpd3Ri/+ (71%) and +/tinG4 (47%) (Fig. 5A). Furthermore, the difference between the survival rates continued to steadily increase thereafter, up to 33% at 42 hours (Figs. 5A and C). Interestingly, a notable enhancement in cardiac function was detected even at zero hours, showing that the genotype with Rpd3 downregulation displays a 16% initial increase of heart rate over the rpd3Ri/+ single transgene control (327 vs. 283 HBM) with persistent augmentation throughout the period of oxidative stress (Figs. 5B-C). Consequently, after 42 hours of oxidative stress the heart rate in flies with Rpd3 downregulation was still 12% higher than the control, possibly correlating improved cardiac function to survival against oxidative stress (Fig. 5C).

In contrast, Rpd3 downregulation within other non-cardiac tissues had no observable impact in either stress resistance or heart function (Figs. 3A and 5C). For example, Rpd3 downregulation in the eye, Rpd3Ri/GMRG4, did not display an increase of survival in the oxidative stress over the Rpd3Ri/ + control during whole time period stressed (Fig. 5C). After 24 hours, the controlled +/GMRG4 and Rpd3Ri/+ cohorts had survival rates of 19% and 65% respectively, yet only 58% of the rpd3Ri/GMRG4 had survived (SFig. 4A). Corroborating this assertion, heart rate was also reflective of being in between both controls; at 24 hours there was a 12% increase in heart rate over the +/GMRG4 genotype, but a 4% decrease over the Rpd3Ri/+ genotype (SFig. 4B). Finally, we compared the differences between heart-specific and eye-specific Rpd3 downregulation through the common control genotype, Rpd3Ri/+ (Fig. 5C). During the oxidative stress, the comparison showed that heart-specific Rpd3 downregulation provides an average enhancement of 13% in cardiac activity, with a gradual increase in oxidative stress resistance up to a maximum 33%. In contrast, eye-specific Rpd3 downregulation averaged only −0.1% and −1.3% changes, respectively (Fig. 5C).

An assessment of Rpd3 downregulation in non-cardiac regions (Figs. 3A and 5C) suggests that reduced expression of the *rpd3* gene translates to enhanced stress survival and cardiac function only when it is specifically targeted at the heart. To validate this assertion, the heart failure and recovery assays were performed via the heart pacing protocol as described by Wessells *et al*. (2004) [10] with applying a sustained 80V electric shock during 30 seconds. Those cardiac functions were measured after a 24 hour treatment of oxidative stress in order to characterize how the cardiac function and stress resistance are related (Figs. 5D-F). At 24 hours point, a survival rate of heart-specific Rpd3 downregulation (Rpd3Ri/tinG4) revealed 19% increase over the control (Rpd3Ri/+ in Fig. 5D). In contrast, eye-specific Rpd3 downregulation (Rpd3Ri/GMRG4) had no effect from the controls (Fig. 5D). It was found that the heart-specific Rpd3 downregulation showing higher stress survivalship yields 14% lower heart failure rate at 4 minutes after electric shock, compared to the control (Rpd3Ri/+ in Fig. 5E). At the 0 and 2 minute points, the heart failure rates were also lower than the control (14% and 18%, respectively). The eye-specific Rpd3 downregulation (Rpd3Ri/GMRG4), however, exhibited only −0.6% change in heart failure (Fig. 5E). A heart recovery rate from heart failure at 0 minute until 4 minutes after electric shock was increased by 18% with the heart-specific Rpd3 downregulation (Fig. 5F). Even in other time periods (0 - 2 min and 2 - 4 min), the higher rates of heart recovery were still observed (14% and 13%, respectively). In a trial that the oxidative stress resistance was not enhanced, interestingly, improved cardiac functions also was not detected (data not shown). All together, these results suggest a positive and exclusive correlation between heart-specific *rpd3* expression and cardiac function/stress tolerance.

### Heart-specific Rpd3WT upregulation decreases both stress resistance and cardiac function, but the no-phosphorylated mutant Rpd3ADA restores them

**Figure 6:**
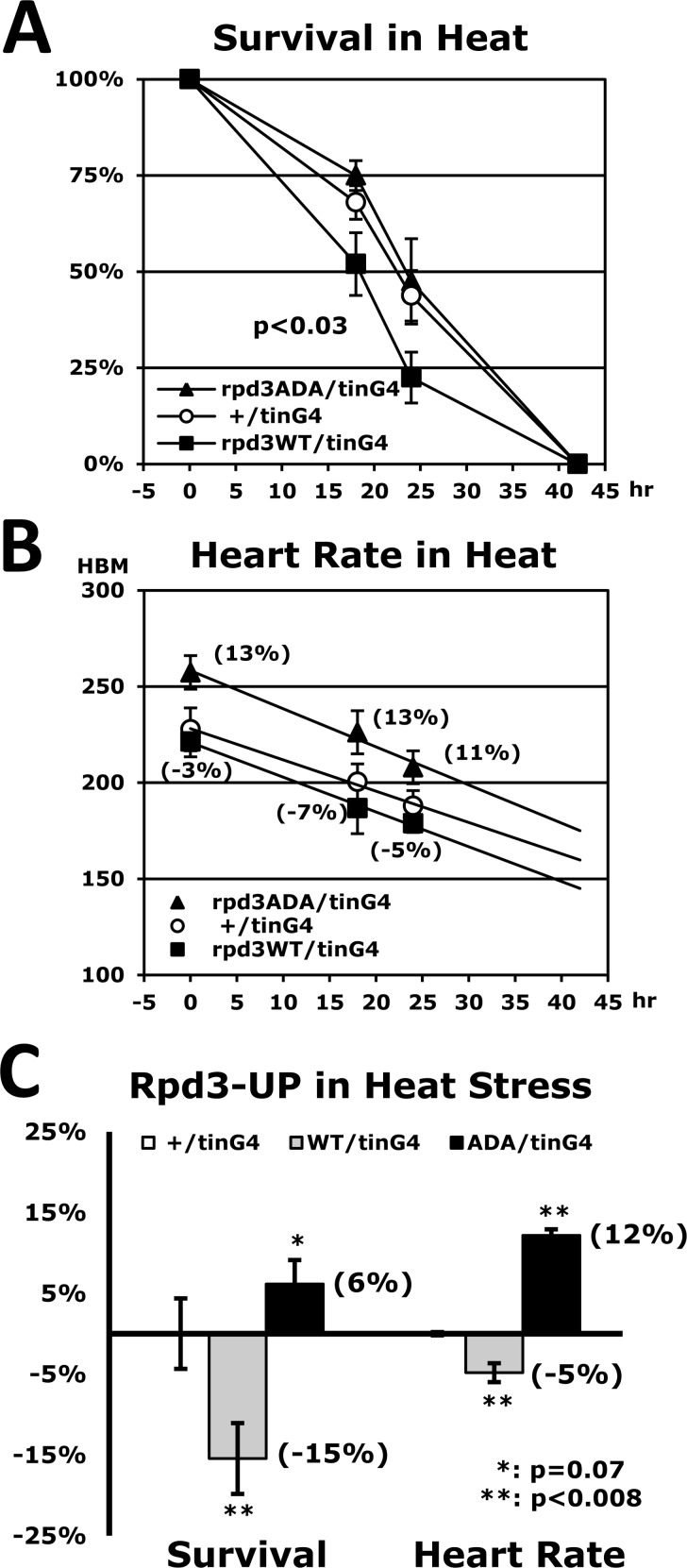
Stress resistance and heart function are reduced by heart-specific Rpd3 upregulation (**A**) Heat-stress survivorship of flies with heart-specific Rpd3 upregulation of wild-type (WT), and non-phosphorylated mutant (ADA: S419A/S421A). The p-value (p<0.03, log-rank test) between +/tinG4 and rpd3WT/tinG4 indicates that heat resistance is significantly diminished with increased *rpd3WT* expression. The p-value between rpd3WT/tinG4 and rpd3ADA/tinG4 shows a more significant difference (p<0.0001). (**B**) The corresponding heart rates during the aforementioned heat stress test (**A**) across the same time points (0, 18, and 24 hours). At each time point, more than 20 heart rates were measured. (**C**) Change of survivorship and heart rates of heart-specific Rpd3 upregulation under heat stress. Changed percentages (parenthesis) of the median survival time of Rpd3WT (or ADA) upregulation (5 independent experiments) were normalized with that of the common control (+/tinG4). The changes in heart rate between the +/tinG4 and Rpd3 upregulations are represented as the average of three time points (0, 18, and 24 hours) in the heart rate graphs (**B**).

Our findings, which the heart-specific Rpd3 downregulation enhances stress tolerance and cardiac function, let us investigate whether *rpd3* upregulation in the heart causes the opposite effect. Using a UAS-*rpd3WT* transgene (Fig. 6A) the stress tolerance was measured under the heat stressor. The heart-specific Rpd3WT upregulation (rpd3WT/tinG4), unsurprisingly, revealed a 15% decrease of the median survival time (hour) in resistance against the heat (Figs. 6A and C) when compared to the control (+/tinG4). Similarly, the heart rate in cardiac function was also reduced by an average of 5% in the different time points of a heat-stressed period (Figs. 6B-C). All assays were statistically significant (P < 0.007), which indicate that the heart-specific Rpd3 upregulation decreases both stress resistance and cardiac function.

Having established that differential levels of rpd3 expression in the heart modulate stress resistance and cardiac function, we further investigated whether the enzymatic activity of Rpd3 is the responsible mechanism for such enhancement. It was reported that the phosphorylation of histone deacetylase 1 (HDAC1), the mammalian homologue of the Rpd3 gene, promotes enzymatic activity and complex formation in mammals [14-16]. The phosphorylation at Rpd3 S419/S421 sites is evolutionarily conserved and known to promote mammalian HDAC1 activity. Through site-directed mutagenesis, a non-phosphorylated *rpd3ADA* clone (S419A/S421A) was produced from the *rpd3WT* construct and the stability of Rpd3ADA protein was similar to the Rpd3WT levels (data not shown).

Interestingly, we found that the impaired stress resistance induced by heart-specific Rpd3WT upregulation disappears when the *rpd3ADA* clone is upregulated instead of *rpd3WT* (Fig. 6A), indicating that phosphorylated Rpd3 in the heart functions as a primary regulator of stress resistance. This heart-specific Rpd3ADA upregulation (rpd3ADA/tinG4) also restored the reduced heart rate caused by heart-specific Rpd3WT upregulation (Fig. 6B). Surprisingly, stress resistance and heart rate in the rpd3ADA/tinG4 genotype were higher than those of the common control +/tinG4 (Fig. 6C), suggesting a possibility that the Rpd3ADA protein competes with endogenous Rpd3WT in the heart and therefore interferes with the function of Rpd3WT in the regulation of stress resistance and cardiac activity.

### Lifespan is extended when Rpd3 is downregulated in a heart continuously during aging

**Figure 7:**
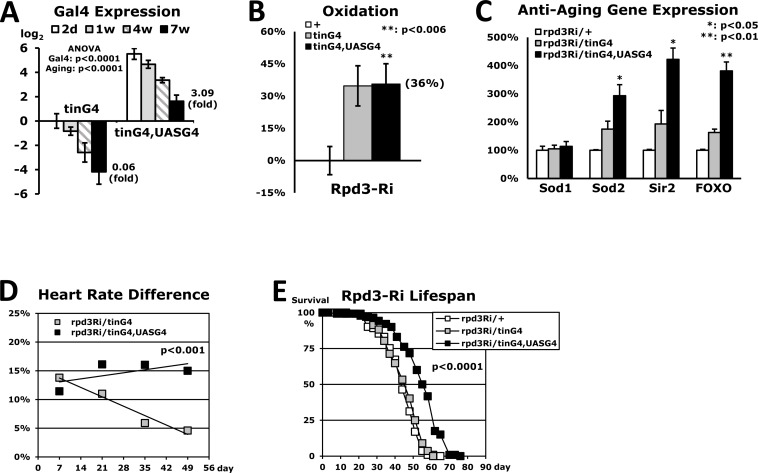
Extended lifespan in flies with heart-specific Rpd3 downregulation (**A**) Heart-specific Gal4 expression levels during aging. The Gal4 expression was measured at the 2-day, 1-week, 4-week, and 7-week time points using the whole body's total RNA of flies with heart-specific Rpd3 downregulation (rpd3Ri/tinG4 and rpd3Ri/tinG4,UASG4). The Gal4 expression of the 2-day old rpd3Ri/tinG4 flies is set as log_2_ = 0 and other Gal4 expressions are compared from 3 - 5 independent RT-PCR experiments. (**B**) Oxidation stress assay of rpd3Ri/tinG4,UASG4 flies, which was normalized with the common control rpd3Ri/+ from five independent experiments. The data of rpd3Ri/tinG4 in Fig. 3A was indicated together for the comparison. (**C**) Differential expression of anti-aging genes in rpd3Ri/tinG4,UASG4 flies, which was obtained by comparison to the 2-day old rpd3Ri/tinG4 male flies (5 - 9 trials). The data was standardized against the common rpd3Ri/+ control (100%) in Fig. 4A. (**D**) Cardiac function throughout the lifespan of flies with heart-specific Rpd3 downregulation, of which heart rates were measured at 1, 3, 5 and 7-week time points. The percent changes in heart rate were represented from a comparison to the single transgene control (0%) at each time point (30 - 100 counting of heart rates). (**E**) The lifespan among adult male flies between the common control (rpd3Ri/+) and heart-specific Rpd3 downregulation (rpd3Ri/tinG4 or rpd3Ri/tinG4,UASG4).

Downregulating the *rpd3* gene in the whole body enhanced both stress resistance and lifespan in the *rpd3*−/+ and rpd3Ri/armG4 flies (Fig. 1 and SFig. 1) [3]. However, the flies that downregulated *rpd3* expression in the heart (rpd3Ri/tinG4) displayed no significant improvement of lifespan (Fig. 7E) in spite of showing higher stress resistance (Fig. 2). Additionally, the improvement of cardiac function in young age diminished progressively throughout the lifespan (SFig. 6A and Fig. 7D). Further experiments revealed that there was insufficient Gal4 expression from the *tinman*-Gal4 transgene in older aged flies to downregulate *rpd3* expression in the heart at effective levels (Fig. 7A). Therefore, a recombinant strain of tinG4 and UASG4 was introduced, in which Gal4 expression can be auto-amplified in the heart. At the age of 2-days, the tinG4,UASG4 flies increased the Gal4 expression up to 46-fold compared to the flies with only the tinG4 transgene (Fig. 7A). This tinG4,UASG4 driver was confirmed to display stronger UAS-GFP signal in the heart than the tinG4 driver (SFig. 5). Oxidative stress resistance of tinG4,UASG4 flies was still 36% higher from the common rpd3Ri/+ control (Fig. 7B) and interestingly, the expression of multiple anti-aging genes were more increased with further downregulation of the heart-specific *rpd3* gene (Fig. 7C). During aging, the tinG4,UASG4 flies also showed a gradual decline in Gal4 expression (Fig. 7A). However, the Gal4 expression of tinG4,UASG4 flies at 7-week old was still higher than that of tinG4 flies at 2-day old by 3.1 fold (Fig. 7A). In addition, the cardiac function improved in young age of the tinG4,UASG4 strain was constantly sustained throughout the lifespan (SFig. 6B and Fig. 7D). Continual heart-specific downregulation of Rpd3 during aging, finally, extended the lifespan up to 24% (Fig. 7E), revealing that the Rpd3 in heart influences a variety of anti-aging mechanism, in turn increasing stress resistance and augmenting lifespan (Fig. 7).

## DISCUSSION

Our data showed that decreased Rpd3 expression in *Drosophila* has a benefit for stress resistance against the environment. To downregulate the *rpd3* gene in whole body, we approached two ways using the heterozygous *rpd3* mutant (P{PZ}rpd3[04556]/+) and the UAS/Gal4 system (rpd3Ri/armG4) (Fig. 1). Both flies showed higher survivorship under oxidative stress compared to the control flies. However, the flies differed in increased survivorship percent (*rpd3*−/+: 31% and rpd3Ri/armG4: 22% in Figs. 1C and F). Considering that the downregulation yield of the *rpd3* gene was different between the two approaches (rpd3−/+: 54% and rpd3Ri/armG4: 40% in Figs. 1A and D), it is possible that more downregulation of the *rpd3* gene may induce higher resistance to stress. In the heart-specific Rpd3 downregulation, a similar pattern was observed between the rpd3Ri/tinG4 and rpd3RiS/tinG4 flies (Fig. 3). The 21bp target sequence of rpd3RiS transgene was less effective at *rpd3* downregulation compared to the 482bp sequences of rpd3Ri transgene when tested in the whole body (data not shown). Thus, the rpd3RiS/tinG4 flies showed a 23% increase in survivorship (Fig. 3C) compared to a 35% increase in rpd3Ri/tinG4 flies (Fig. 3A). Those data let us speculate that the content of *rpd3* downregulation determines the consequent stress-resistance enhancement.

It was found that heart-specific Rpd3 downregulation systemically increases expression of anti-aging genes such as Sod2 and dFOXO (Fig. 4A). It was also shown that more downregulation of the *rpd3* gene in a heart induces higher expression of anti-aging genes (Fig. 7C). This may provide an explanation of how Rpd3 downregulation in the heart enhances stress resistance mechanism, particularly since dFOXO is considered to activate *sod2* gene [30]. In response to cellular stresses, such as nutrient deprivation or increased levels of reactive oxygen species, dFOXO is activated and inhibits growth through acting on target genes such as *Thor* (d4E-BP) [31]. As a translational repressor, 4E-BP activity is shown to be critical for survival under dietary restriction and oxidative stress, and is linked to lifespan [32, 33]. This dFOXO/4E-BP signaling is also revealed to play a key role in the coordination of organismal and tissue aging through an organism-wide regulation of proteostasis in response to muscle aging [34]. Interestingly, this *Drosophila* forkhead transcription factor (dFOXO) activates d4E-BP transcription [31, 32], which is upregulated under stressed conditions [32]. Consistent with increased *foxo* expression in flies with heart-specific Rpd3 downregulation (Fig. 4A), our data also showed that *Thor* was significantly upregulated with heart-specific Rpd3 downregulation (Fig. 4B). When induced by stress, fat body antimicrobial peptide (AMP) genes are activated in response to nuclear dFOXO activity [35]. Upregulation of both *foxo* and *DptB* (one of target AMP) genes in heart-specific Rpd3 downregulation (Figs. 4A-B) illustrates that Rpd3 downregulation in the heart modulates gene expression in other tissues such as fat body for stress adaption. One possible mechanism of this modulation is that heart-specific Rpd3 downregulation produces secreted proteins through Rpd3 deacetylase activity from heart, which thus regulates gene expression in other tissues.

A positive correlation between stress resistance and lifespan extension was shown in several long-lived mutant flies [6-9]. Previous findings have also suggested that enhanced stress resistance may extend lifespan in *Drosophila* [36, 37]. Our data indicated that downregulating the *rpd3* gene in the whole body or heart enhances both stress resistance and lifespan with improved cardiac function. However, insufficient heart-specific Rpd3 downregulation in older aged flies failed to prolong lifespan or improve cardiac condition (Fig. 7), implying that throughout lifetime, Rpd3 in the heart influences both cardiac function and lifespan. Currently, although a conclusion of whether improved cardiac function from heart-specific Rpd3 modulation directly impacts longevity mechanism cannot yet be made, it is reported that enhanced cardiac capability could extend the lifespan of *Drosophila* [38].

## METHODS

### Fly genotypes and cross

The heart-specific *tinC*∆*4*-Gal4 (tinG4) flies were kindly provided by Rolf Bodmer [20]. The *rpd3*−/+ (P{PZ}rpd3[04556]), UAS-*rpd3*-dsRNAi, UAS-*loco*-dsRNAi, UAS-*sir2*, and Gal4 driver flies were obtained from Bloomington *Drosophila* stock center except for the UAS-*loco*-dsRNAi flies (V110275, Vienna *Drosophila* RNAi Center) [17]. The lab stock strain *y^1^ w^1^* (Bloomington) were used as wild-type control and the flies obtained outside were six times isogenized with *y^1^ w^1^* before the stress resistance and aging tests. Virgin flies were collected from a bottle in which larval density was controlled in standard cornmeal medium, and were used for all fly experiments including stress response and aging studies.

### Constructs and RT-PCR

To construct rpd3WT and rpd3ADA transgenes (Fig. 6), the *rpd3* wild-type cDNA (1,563 bp) was cloned into the pCRII-TOPO vector (Invitrogen) and, from the rpd3WT plasmid the rpd3ADA clone (S419A/S421A) was produced using QuikChange Site-Directed Mutagenesis Kit (Agilent Technologies). After sequencing was completed, the plasmids were subcloned into a XhoI/XbaI digested pUASTattB vector.

To check transcriptional expression of the genes, 5 μg of the total RNA purified from adult flies (TRIzol, Invitrogen) were treated with DNase I (RNase-free, Roche) and used to produce oligo dT-primed cDNAs (SuperScript II RT, Invitrogen), which were then used as templates for quantitative real-time PCR [39]. The *rp49* gene was used as an internal reference for normalizing the quality of total RNAs. Real-time PCR was performed with SYBR green using ABI7300 Real-time PCR Instrument (Applied Biosystems). Expressional fold of the various genes were determined by the comparative C_T_ method (ABI Prism 7700 Sequence Detection System User Bulletin #2, Applied Biosystems).

### Stress response and aging assays

To measure responses for each of the three stressors (starvation, oxidation and heat), 100 newborn male flies (20 flies per vial) were kept on standard cornmeal medium at 25°C for two days [6, 40]. For the starvation stress-response assay, these two-day-old flies were transferred into clean vials (2.5 × 9.3 cm) that contain two-filter circle (2.4-cm diameter, Fisher Scientific) soaked with 300 μl of distilled water. Then, the vials were maintained at 25°C under moist conditions with 100 μl of distilled water added every 12 hours. For the oxidative stress assay, the two-day-old adult flies were starved for 6 hours at 25°C as described above. Then after the 6 hours starvation period, the flies were transferred to new vials containing two filter circles wetted with 300 μl of 20 mM methyl viologen hydrate (Paraquat, Fisher Scientific) in a 5% sucrose solution and maintained at 25°C. For the heat test, the two-day-old flies were transferred into vials with standard cornmeal medium and were kept in a 37°C incubator with 30% humidity. The survival number for each of the experiment was manually counted with four hour intervals. To test for females, five-day-old adult flies were used following the same procedure for each of the assays. For the aging test, 200 virgin flies (20 flies per vial) were counted and transferred to fresh standard cornmeal vials every three to four days [6, 40].

### Heart rate assay

To measure the heart rate, the flies in a FlyNap chamber were anesthetized with 40 μl of FlyNap (Carolina) for 3 minutes [41]. Then, the flies were placed on a microscopic slide with tape on it and the wings of the flies were brushed off to the side so the heart was clearly visible. Using an Olympus microscope, three videos consisting of 20 seconds for each fly were recorded and the heart rate was manually counted and averaged. For each independent experiment, four to eight flies were used. During aging, this procedure was repeated biweekly from one-week old flies until they became seven-week old flies [10].

### Heart failure and recovery rate assays

To observe heart failure in the flies, a microscope slide with two electrodes on opposite ends was prepared [42]. Conductive electrode jelly was spread between the two electrodes, leaving a minuscule gap in the middle to provide an electric shock through the flies. Flies of several genotypes were anesthetized with 40 μl of FlyNap for three minutes and then placed onto the microscope slide oriented so that the head and abdomen of the fly are facing opposite electrodes and are perpendicular to the gap in the conductive jelly. The wings of the flies were pushed onto the side of the flies on the conductive jelly to get a better view of the heart. After a maximum of 12 flies were placed onto one microscope slide, the slide was placed under the OLYMPUS microscope to observe the heartbeat. Then, the flies were electrically paced with a Grass SD9 stimulator at 80V, 6Hz, 0 Delay, 30ms Duration for 30 seconds. Cardiac arrest was measured at 0, 2 and 4 minutes after the pacing stimulation. Heart failure rate was calculated, among all the flies by counting the flies in a state of cardiac arrest until 4 minutes have passed. To obtain heart recovery rate, the amount of flies that restores their heart beat at 4 minutes after being in a state of cardiac-arrested at 0 minute was measured [10].

## SUPPLEMENTARY FIGURES



## References

[1] Meier K, Brehm A (2014). Chromatin regulation: how complex does it get?. Epigenetics : official journal of the DNA Methylation Society.

[2] Fitzsimons HL, Scott MJ (2011). Genetic modulation of Rpd3 expression impairs long-term courtship memory in Drosophila. PLoS One.

[3] Rogina B, Helfand SL, Frankel S (2002). Longevity regulation by Drosophila Rpd3 deacetylase and caloric restriction. Science.

[4] Kim S, Benguria A, Lai CY, Jazwinski SM (1999). Modulation of life-span by histone deacetylase genes in Saccharomyces cerevisiae. Mol Biol Cell.

[5] Kang HL, Benzer S, Min KT (2002). Life extension in Drosophila by feeding a drug. Proc Natl Acad Sci USA.

[6] Lin YJ, Seroude L, Benzer S (1998). Extended life-span and stress resistance in the Drosophila mutant methuselah. Science.

[7] Clancy DJ, Gems D, Harshman LG, Oldham S, Stocker H, Hafen E, Leevers SJ, Partridge L (2001). Extension of life-span by loss of CHICO, a *Drosophila* insulin receptor substrate protein. Science.

[8] Vermeulen CJ, Loeschcke V (2007). Longevity and the stress response in Drosophila. Experimental gerontology.

[9] Lin YR, Parikh H, Park Y (2011). Loco signaling pathway in longevity. Small GTPases.

[10] Wessells RJ, Fitzgerald E, Cypser JR, Tatar M, Bodmer R (2004). Insulin regulation of heart function in aging fruit flies. Nat Genet.

[11] Ocorr K, Akasaka T, Bodmer R (2007). Age-related cardiac disease model of Drosophila. Mech Ageing Dev.

[12] Winnik S, Auwerx J, Sinclair DA, Matter CM (2015). Protective effects of sirtuins in cardiovascular diseases: from bench to bedside. Eur Heart J.

[13] Haberland M, Montgomery RL, Olson EN (2009). The many roles of histone deacetylases in development and physiology: implications for disease and therapy. Nature reviews Genetics.

[14] Galasinski SC, Resing KA, Goodrich JA, Ahn NG (2002). Phosphatase inhibition leads to histone deacetylases 1 and 2 phosphorylation and disruption of corepressor interactions. The Journal of biological chemistry.

[15] Pflum MK, Tong JK, Lane WS, Schreiber SL (2001). Histone deacetylase 1 phosphorylation promotes enzymatic activity and complex formation. J Biol Chem.

[16] Pluemsampant S, Safronova OS, Nakahama K, Morita I (2008). Protein kinase CK2 is a key activator of histone deacetylase in hypoxia-associated tumors. International journal of cancer Journal international du cancer.

[17] Lin YR, Kim K, Yang Y, Ivessa A, Sadoshima J, Park Y (2011). Regulation of longevity by regulator of G-protein signaling protein, Loco. Aging Cell.

[18] Baldal EA, Baktawar W, Brakefield PM, Zwaan BJ (2006). Methuselah life history in a variety of conditions, implications for the use of mutants in longevity research. Exp Gerontol.

[19] Brand AH, Perrimon N (1993). Targeted gene expression as a means of altering cell fates and generating dominant phenotypes. Development.

[20] Qian L, Bodmer R (2009). Partial loss of GATA factor Pannier impairs adult heart function in *Drosophila*. Hum Mol Genet.

[21] Hotamisligil GS (2006). Inflammation and metabolic disorders. Nature.

[22] Hull-Thompson J, Muffat J, Sanchez D, Walker DW, Benzer S, Ganfornina MD, Jasper H (2009). Control of metabolic homeostasis by stress signaling is mediated by the lipocalin NLaz. PLoS Genet.

[23] Giannakou ME, Goss M, Junger MA, Hafen E, Leevers SJ, Partridge L (2004). Long-lived Drosophila with overexpressed dFOXO in adult fat body. Science.

[24] Hwangbo DS, Gershman B, Tu MP, Palmer M, Tatar M (2004). Drosophila dFOXO controls lifespan and regulates insulin signalling in brain and fat body. Nature.

[25] Whitaker R, Faulkner S, Miyokawa R, Burhenn L, Henriksen M, Wood JG, Helf SL (2013). Increased expression of Drosophila Sir2 extends life span in a dose-dependent manner. Aging (Albany NY).

[26] Tatar M, Kopelman A, Epstein D, Tu MP, Yin CM, Garofalo RS (2001). A mutant *Drosophila* insulin receptor homolog that extends life-span and impairs neuroendocrine function. Science.

[27] Ekengren S, Hultmark D (2001). A family of Turandot-related genes in the humoral stress response of *Drosophila*. Biochem Biophys Res Commun.

[28] Landis GN, Abdueva D, Skvortsov D, Yang J, Rabin BE, Carrick J, Tavare S, Tower J (2004). Similar gene expression patterns characterize aging and oxidative stress in *Drosophila melanogaster*. Proc Natl Acad Sci USA.

[29] Ekengren S, Tryselius Y, Dushay MS, Liu G, Steiner H, Hultmark D (2001). A humoral stress response in *Drosophila*. Current biology : CB.

[30] Curtis C, Landis GN, Folk D, Wehr NB, Hoe N, Waskar M, Abdueva D, Skvortsov D, Ford D, Luu A, Badrinath A, Levine RL, Bradley TJ (2007). Transcriptional profiling of MnSOD-mediated lifespan extension in *Drosophila* reveals a species-general network of aging and metabolic genes. Genome Biol.

[31] Junger MA, Rintelen F, Stocker H, Wasserman JD, Vegh M, Radimerski T, Greenberg ME, Hafen E (2003). The Drosophila forkhead transcription factor FOXO mediates the reduction in cell number associated with reduced insulin signaling. J Biol.

[32] Tettweiler G, Miron M, Jenkins M, Sonenberg N, Lasko PF (2005). Starvation and oxidative stress resistance in Drosophila are mediated through the eIF4E-binding protein, d4E-BP. Genes Dev.

[33] Zid BM, Rogers AN, Katewa SD, Vargas MA, Kolipinski MC, Lu TA, Benzer S, Kapahi P (2009). 4E-BP extends lifespan upon dietary restriction by enhancing mitochondrial activity in Drosophila. Cell.

[34] Demontis F, Perrimon N (2010). FOXO/4E-BP signaling in Drosophila muscles regulates organism-wide proteostasis during aging. Cell.

[35] Becker T, Loch G, Beyer M, Zinke I, Aschenbrenner AC, Carrera P, Inhester T, Schultze JL, Hoch M (2010). FOXO-dependent regulation of innate immune homeostasis. Nature.

[36] Parkes TL, Elia AJ, Dickinson D, Hilliker AJ, Phillips JP, Boulianne GL (1998). Extension of *Drosophila* lifespan by overexpression of human SOD1 in motorneurons. Nat Genet.

[37] Chavous DA, Jackson FR, O'Connor CM (2001). Extension of the *Drosophila* lifespan by overexpression of a protein repair methyltransferase. Proc Natl Acad Sci USA.

[38] Kaushik G, Spenlehauer A, Sessions AO, Trujillo AS, Fuhrmann A, Fu Z, Venkatraman V, Pohl D, Tuler J, Wang M, Lakatta EG, Ocorr K, Bodmer R (2015). Vinculin network-mediated cytoskeletal remodeling regulates contractile function in the aging heart. Sci Transl Med.

[39] Park SW, Oh H, Lin YR, Park Y (2010). MSL *cis*-spreading from *roX* gene up-regulates the neighboring genes. Biochem Biophys Res Commun.

[40] Kim K, Lin YR, Park Y (2010). Enhancement of stress resistances and downregulation of Imd pathway by lower developmental temperature in *Drosophila melanogaster*. Exp Gerontol.

[41] Wessells RJ, Bodmer R (2004). Screening assays for heart function mutants in Drosophila. BioTechniques.

[42] Ocorr K, Vogler G, Bodmer R (2014). Methods to assess Drosophila heart development, function and aging. Methods.

