# Supplementary material for: Heart-specific Rpd3 downregulation enhances cardiac function and longevity
Source: Aging (Albany NY). 2015 Sep 21;7(9):648–60. doi: 10.18632/aging.100806 (PMC4600623; doi:10.18632/aging.100806)
Supplement: Supplementary file 1 [file aging-07-648-s001.pdf]

SUPPLEMENTARY FIGURES

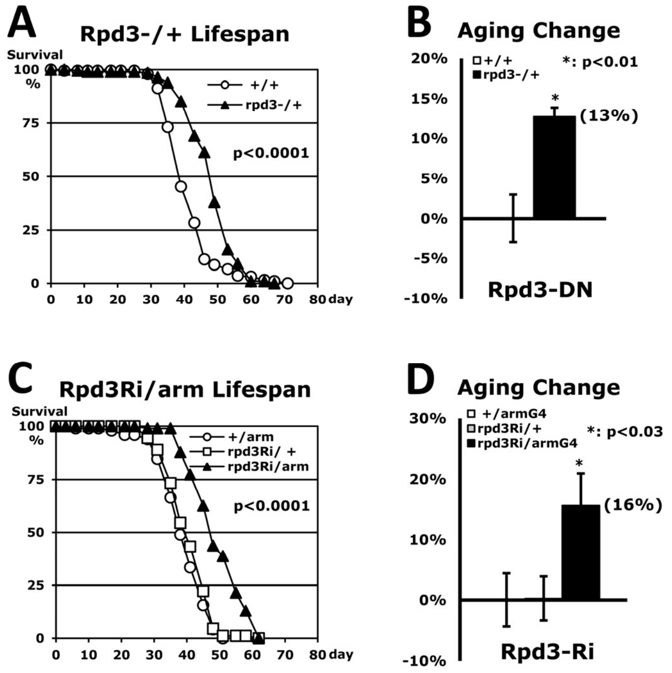

SFigure 1. Lifespan extension of Rpd3 downregulation. (**A**) The aging test comparing wild-type and *rpd3* heterozygote mutant (*rpd3*-/+), using adult male flies. (**B**) The increased percentage of the *rpd3* mutant's mean lifespan is indicated as mean ± SEM normalized with respect to wild-type (39.7 days). This percentage was calculated from the lifespan curves (**A**) of two independent experiments. (**C**) The aging test between two single transgene controls (+/armG4, rpd3Ri/+) and the double transgene experimental (rpd3Ri/armG4) male flies. The p-values (log-rank test) of the two lifespan curves (**A** and **C**) indicate that lifespan increases with a decrease in *rpd3* expression. (**D**) Increased percentage of the mean lifespan of rpd3Ri/armG4 is indicated as mean ± SEM normalized by the +/armG4, which was calculated from the lifespan curves (**C**) of four independent experiments.

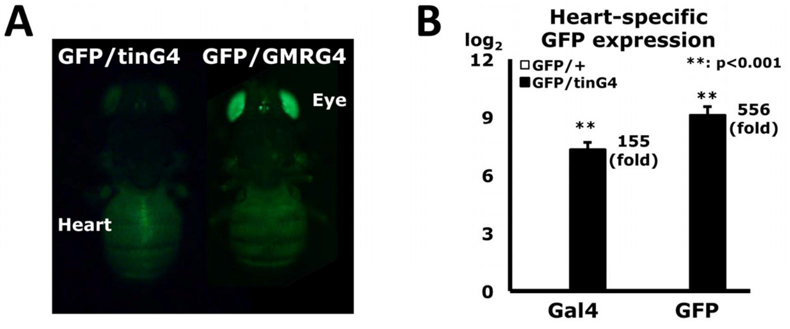

SFigure 2. Heart-specific Gal4 expression (tinG4). (**A**) **Left:** Heart-specific (tinman) GFP expression (UAS-GFP/tin-Gal4), illuminating the long heart tube from the posterior abdomen. **Right:** Eye-specific GFP expression (UAS-GFP/GMR-Gal4), used as a control. (**B**) Heart-specific expression levels of Gal4 and the target GFP genes from the whole body's total RNA between UAS-GFP/+ and UAS-GFP/tin-Gal4 using RT-PCR.

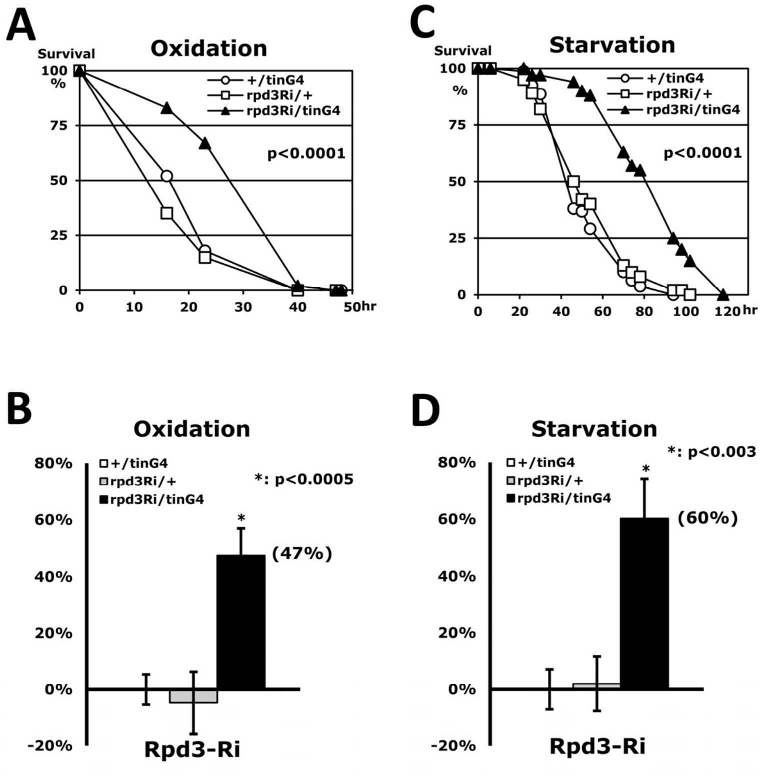

SFigure 3. Stress resistance induced by heart-specific Rpd3 downregulation in female flies. (**A** and **C**) The response to oxidation (**A**) and starvation (**C**) stressors using 5-day-old female flies. The flies with heart-specific *rpd3* downregulation (rpd3Ri/tinG4) were compared to the single transgene controls (+/tinG4 and rpd3Ri/+). The survival curves show that the p-values for both stressors (**A** and **C**) were statistically significant (P < 0.0001), indicating that resistances to both stressors are enhanced also in females with heart-specific *rpd3* downregulation. (**B** and **D**) The percent change in the median survival time of flies for the stresses is indicated as mean ± SEM, which was calculated from the stress response curves of 5 - 7 independent experiments. Parenthesis: percent change in heart specific Rpd3 downregulation as compared to the +/tinG4 single transgene control.

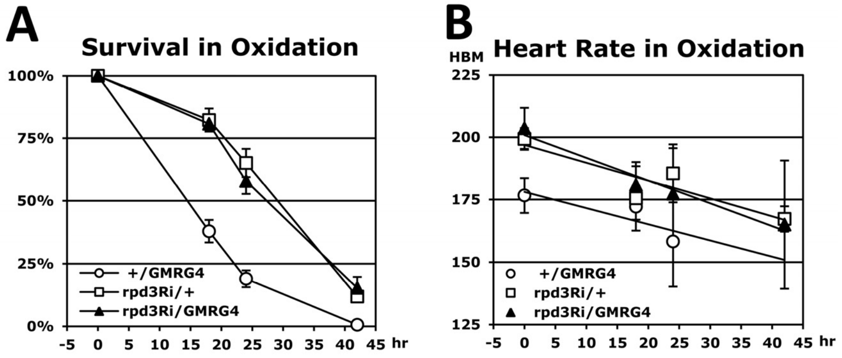

SFigure 4. Oxidative stress resistance and heart function unaffected by eye-specific Rpd3 downregulation. (**A**) The survival curve for oxidative stress between 2-day-old male flies of single transgene controls and eye-specific Rpd3 downregulation (rpd3Ri/GMRG4), which was made with the mean ± SEM over four independent assays. (**B**) The concurrent heart rate during the oxidative stress above (**A**). The heart rate of +/GMRG4 at 42 hours could not be measured due to no survivorship in (**A**). The resultant change in heart rate between the rpd3Ri/+ and rpd3Ri/GMRG4 revealed that the heart rate is not changed by reduced *rpd3* expression in the eye throughout the oxidative stress.

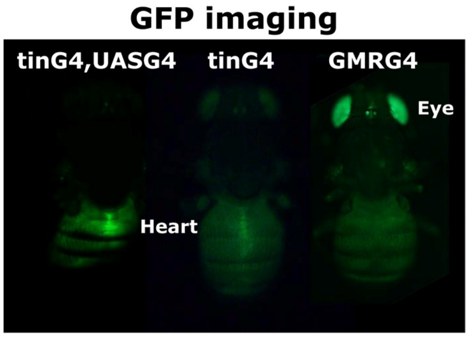

SFigure 5. GFP imaging with heart-specific Gal4 expression (tinG4 and tinG4, UASG4).

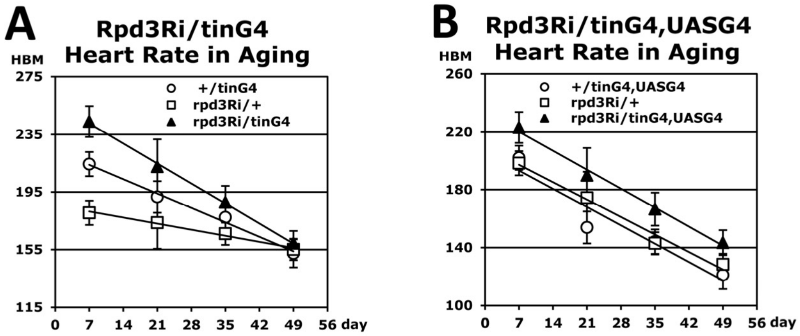

SFigure 6. Heart rates of flies with heart-specific Rpd3 downregulation. (**A-B**) Heart rates of rpd3Ri/tinG4 (**A**) and rpd3Ri/tinG4,UASG4 (**B**) were measured at the 1, 3, 5 and 7-week time points during the aging experiments in Fig. 7E. The 30 - 100 of heart rates were counted at each age and averaged for mean ± SEM over 3 - 9 independent assays.
